# Historical perspective: the social determinants of disease – some blossoms

**DOI:** 10.1186/1742-5573-2-4

**Published:** 2005-06-02

**Authors:** Michael Marmot

**Affiliations:** 1 University College London, Department of Epidemiology & Public Health Gower Street Campus, 1-19 Torrington Place, London WC1E 6BT, UK

## Introduction

I had two great teachers in epidemiology: Len Syme and Geoffrey Rose. One had his thinking shaped by the insights of Durkheim, a great sociologist; the other by Pickering, a great hypertension specialist. One helped lay the foundations for social epidemiology; the other, if heeded, could change the way we think about public health. Both came to the conclusion that society mattered for health and that one could not understand the social rate of disease simply by studying individuals. The story of laying the foundations for social epidemiology is engagingly set out in the accompanying paper by Len Syme.[1] Geoffrey Rose's credo is beautifully articulated in his last book.[2]

## Scientist as teacher and mentor

Syme's memoir of his vital role in the origins of social epidemiology is characteristically modest. He alludes to his students but his paper does not convey the half of it. I have never come across anyone in the academic world who had quite the powerful influence on students that Syme did. With cohort after cohort of students he developed special relationships, he with them and they with him. He was stimulating, challenging, encouraging, sensitive, hands off when appropriate, guiding when needed, critical when constructive, praising when spirits required i.e. more or less always.

I suspect he had few failures, in part because there was "assortative mating" – a matching of teacher and student that had the right chemistry, but also because he was simply so good at bringing the best out of people and then helping them with their subsequent careers. Not all students came under his magic spell – some had different interests and sought different mentors – but my guess is that he helped everyone who came into his orbit.

For some of us the relationship, begun at Berkeley, became one of life long friendship and collegiality. I knew how much he influenced me while I was at Berkeley as a graduate student and young teacher, and then, having left, I discovered all over again how many of my "original" thoughts had their origins in Berkeley.

Syme encouraged interdisciplinary work. He taught the importance of asking the right question. We were encouraged to seek insight wherever it may be found – and on the Berkeley campus there was no shortage of insight. And he showed what it meant to consider social causes. I touch on each of these below.

## Analysis

### 'Doctors have no special insight on causes of ill-health'

That was really shocking. How dare he? But Syme did say that to his students. Like most physicians I assumed that only doctors could understand the causes of illness. It was we, after all, who laid claim to some understanding of biology and pathology. Giving primacy to biology betrayed a classic misunderstanding of the notion of cause. It may be useful to think of accumulation of lipid in the endothelium of coronary arteries as "the cause" of coronary heart disease – a good pathologist's perspective on cause – but what causes the accumulation of lipid? I now use Geoffrey Rose's phrase: the causes of the causes[3] (it has less tendency to frighten the horses than other terms that I still use) but my first introduction to such thinking was from Syme.

Syme's argument was that the reading of a medical textbook conferred no unique insight into the social causes of disease. Just as medical training, or its equivalent, may be necessary to understand the mechanisms of disordered pathology so is understanding of society necessary to understand the social causes of illness. He was not a social scientist who considered biology unimportant and social science paramount. Both were needed. That lesson has stayed with me.

### 'Three questions'

Actually four. There was a meta-level. Syme taught that the first, perhaps most important, question was: "what's the question?" This then resolved into three separate questions one should ask about the occurrence of disease:

Why does one group have a higher rate of disease than another?

Why does one individual in a group get sick and another not?

When an individual succumbs to illness what determines which illness s/he gets?

Syme pointed out that most 'current' (1970s but still the case) epidemiological research pursued the second of these questions: the search for risk factors. In the 1970s it was blood pressure and cholesterol. In the 2000s it is C Reactive Protein. The thinking is the same: will a risk factor discriminate those who will get sick from those who won't.

Syme refers in his paper to John Cassel. Both Syme and Cassel were active in pursuing the argument that individuals' risks of disease seemed to vary in parallel, i.e., people at risk of one disease seemed to be at risk of others that, according to conventional wisdom, had different pathology. Hence two different questions (the second and third in the above list): why did an individual get sick and what determined the particular disease from which he suffered. Syme and Lisa Berkman wrote an important review paper: *Social Class, Susceptibility and Sickness*[4]. In it they pointed out that low social class was associated with high risk of a range of apparently unrelated diseases. That is to say, they built on a notion of susceptibility that may relate to more than one illness. It is a notion that makes many uncomfortable in the world of medical research.

The hypothesis of general susceptibility leads to a clash of ideas. One view is that low status people, for example, have a high risk of lung cancer because of smoking; of stomach cancer because of diet; of stroke because of diet; of heart disease because of diet, smoking, and sloth; of renal disease because of infection; of suicide because of ... I'll think of something; and so on. Syme threw out the challenge that there may be a common reason underlying these various ways of becoming ill – one should search that out. Separately, one should enquire why one person got heart disease and another, tragically, took his own life.

Syme spells out how an understanding of Durkheim leads to the other crucial distinction; between enquiring after the causes of variation among individuals and variation among groups. The reason why one young man, in a deprived inner city neighbourhood, gets shot and not another is the individual difference question. The reason why young deprived inner-city men have a higher rate of violent deaths than men in the affluent suburbs is a different sort of question. The answers to these two questions, the first and second of the triad, may not be the same.

### Theory?

Syme is disarming about his 'misguided' counsel to ignore theory. I discovered, on leaving Berkeley for the UK, that the two worst criticisms levelled by social scientists against epidemiologists was that they were positivists and atheoretical. It can't get harsher than that! An extreme form of reaction to lack of theory and positivism is social constructionism. There is no objective reality – all is a social construction.

My first encounter with extreme relativism – no objective truth just better or worse theories – came, at Berkeley, from Paul Feyerabend's philosophy of science seminar.[5] Turning up there was the result of Syme's general encouragement to 'go forth, young man' and explore. Nothing was ruled out. Quite magic! Feyerabend was working on his book, *Against Method*, and his catch phrase was: anything goes. It was simply hubris, said Feyerabend, to think that modern science was in some way better than pre-Socratic philosophy. But, I protested, with modern science, we are eliminating smallpox, and putting men in space. "Simple technology! Judge the science by how it performs according to its own lights," was Feyerabend's response. In other words, just because a theory led to elimination of a disease responsible for a scourge on mankind was, in itself, no proof that it was a better theory than one that was internally consistent. I was only partly convinced.[6]

This is, perhaps, a caricature. But not much. Faced with that sort of theory, one can see why Syme might have sought facts. But if a theory is a way of making sense of observations about the world then, I would argue, Syme did have us groping towards theory. It is true that when I first started to study the social gradient in health in Britain[7] I thought it did not much matter how one classified people. Occupational status, education, income all predicted differences in health and life expectancy. Social position was clearly important. What did it matter if the investigator were a Marxist or a Weberian? Surely theory was beside the point. That said, I did acknowledge that we have theory all the time. It permeates our very observations. Recognising that is not to subside into relativism. It is, however, a call to stand back and ask questions about what we are doing and what it means.

Let me illustrate. Syme suggested that 'control of destiny' was a fundamental influence on disease.[8] Separately Karasek and Theorell had been developing their job strain model.[9,10] They proposed that it was not simply job demands that were important for health, but the imbalance between demands and control. Workers faced with high demands but who had little control were at increased risk of cardiovascular and other diseases. Was that theory? It certainly was a way of making sense of some disconnected thoughts and observations. People, going back to Sir William Osler, thought that high status individuals had more heart attacks than lower status because, among other influences, of the high stress that their exalted positions entailed. Yet, in the 1970's, we showed that in British civil servants, the lower their status the higher the mortality from coronary heart disease.[7] This was not a phenomenon unique to British civil servants but was true of the country as a whole.[11] Did that refute the proposition that stress was an important factor in cardiovascular disease? Karasek and Theorell, and Syme, provided a framework, theory even, for why it did not. Control was likely to be lower in lower status groups. Indeed, that is what we showed.[12]

If anyone is 'doing theory', surely it is philosophers. When I first came to read Amartya Sen's work – Sen is as much philosopher as economist – it was a revelation.[13] He was an empirical economist who examined the theoretical basis of what he did. One of his fundamental contributions was the idea of capabilities. What was important to social inequalities was not so much what one had but what one could do with what one had. This 'theory' provided a way of thinking about the importance of control – it was part of capability, what you could do with what you had. At the same time as Sen was dazzling with his insights, another philosopher and a political scientist, Doyal and Gough, elaborated a theory of human need, in which they argued that autonomy was a fundamental human need that cut across cultures and was therefore a way of rising above relativism.[14] This human needs approach gave me a way of thinking about my own struggle to understand the reasons for the social gradient in health.[15]

This by any other name is theory, and Syme contributed to it actively. He is, once again, too modest about his own role here.

## Conclusion

### Social causes

It is, still, an unusual idea that diseases have social causation and that the remedies for social causation might be social in nature. They need not, of course. Syme spent a good part of his life trying to encourage individual people to change their diets and give up smoking. He came away from that experience claiming that it was too difficult to change individuals. He was going to try something easier: change society. Syme's enduring contribution has been as an advocate for the idea that social processes may be as much causes of illness as are biological processes.

If the remedies of the social causes of health should be social, what should we do? I am now up to my ears in a new Commission on Social Determinants of Health.[3] We are trying to take a social approach to reducing inequalities in health between and within countries. The emphasis is on action. In my own mind, there is a continuous line between Syme's teachings and my own current activities. I blame Len Syme.
